# Over- and under-prescribing, and their association with functional disability in older patients at risk of further decline in Germany – a cross-sectional survey conducted as part of a randomised comparative effectiveness trial

**DOI:** 10.1186/s12877-022-03242-w

**Published:** 2022-07-07

**Authors:** Claudia Salm, Julia Sauer, Nadine Binder, Aline Pfefferle, Mario Sofroniou, Gloria Metzner, Erik Farin-Glattacker, Sebastian Voigt-Radloff, Andy Maun

**Affiliations:** 1 Institute of General Practice / Family Medicine, Faculty of Medicine and Medical Center – University of Freiburg, Elsässerstraße 2n, 79110 Freiburg, Germany; 2 Section of Health Care Research and Rehabilitation Research, Institute of Medical Biometry and Statistics, Faculty of Medicine and Medical Center - University of Freiburg, Hugstetterstraße 49, 79106 Freiburg, Germany; 3 Center for Geriatric Medicine and Gerontology Freiburg, Faculty of Medicine and Medical Center - University of Freiburg, Lehenerstraße 88, 79106 Freiburg, Germany; 4 Institute for Evidence in Medicine, Faculty of Medicine and Medical Center - University of Freiburg, Breisacher Straße 153, 79110 Freiburg, Germany

**Keywords:** inappropriate prescribing, potentially inappropriate medications, potential prescribing omissions, polypharmacy, multimorbidity, functional disability

## Abstract

**Background:**

Older patients at risk of functional decline are frequently affected by polypharmacy. This is associated with a further loss of independence. However, a relationship between functional disability and medications, such as ‘Potentially Inappropriate Medications’ (PIMs) and ‘Potential Prescribing Omissions’ (PPOs), as itemised for (de) prescribing in practice-orientated medication lists, has yet to be established.

**Methods:**

As part of a randomised comparative effectiveness trial, *LoChro*, we conducted a cross-sectional analysis of the association between PIMs and PPOs measured using the ‘Screening Tool of Older Persons’ Prescription Criteria / Screening Tool To Alert to Right Treatment’ (STOPP/START) Version 2, with functional disability assessed using the ‘World Health Organization Disability Assessment Schedule 2.0’ (WHODAS). Individuals aged 65 and older at risk of loss of independence were recruited from the inpatient and outpatient departments of the local university hospital. Multiple linear regression analysis was used to model the potential prediction of functional disability using the numbers of PIMs and PPOs, adjusted for confounders including multimorbidity.

**Results:**

Out of 461 patients, both the number of PIMs and the number of PPOs were significantly associated with an increase in WHODAS-score (Regression coefficients B 2.7 [95% confidence interval: 1.5-3.8] and 1.5 [95% confidence interval: 0.2-2.7], respectively). In WHODAS-score prediction modelling the contribution of the number of PIMs exceeded the one of multimorbidity (standardised coefficients beta: PIM 0.20; multimorbidity 0.13; PPO 0.10), whereas no significant association between the WHODAS-score and the number of medications was seen. 73.5 % (339) of the participants presented with at least one PIM, and 95.2% (439) with at least one PPO. The most common PIMs were proton pump inhibitors and analgesic medication, with frequent PPOs being pneumococcal and influenza vaccinations, as well as osteoporosis prophylaxis.

**Conclusions:**

The results indicate a relationship between inappropriate prescribing, both PIMs and PPOs, and functional disability, in older patients at risk of further decline. Long-term analysis may help clarify whether these patients benefit from interventions to reduce PIMs and PPOs.

## Background

Prescribing appropriately is a major challenge in the medical care of older patients. The impact of polypharmacy, as well as of ‘Potentially Inappropriate Medications’ (PIMs), on adverse events has been widely studied with differing outcomes: polypharmacy, most often defined as the concurrent intake of five or more medications [1], is associated with frailty [2], hospitalisation [3] and mortality [4]. Excessive polypharmacy, that is, the intake of ten or more drugs, shows an association with reduced quality of life [5, 6]. Yet, PIMs reveal a less concise association with adverse health outcomes [7, 8]. Major instruments in identifying PIMs are the Beers Criteria [9] and the PIM-identifying criteria of the Screening Tool of Older Persons’ Prescription Criteria / Screening Tool To Alert to Right Treatment’ (STOPP/START) Version 2 [10], often referred to as ‘STOPP criteria’. A retrospective cohort study of more than 170,000 older patients in the United States confirmed the Beers Criteria [9] as well as the STOPP criteria [10] as having a modest capacity to predict adverse drug events and hospitalisations, with the STOPP Criteria performing slightly more accurately [11]. In a two-year prospective cohort study the prescription of more than one PIM was associated with decreased health-related quality of life [12]. Furthermore, polypharmacy with at least one medication listed in the Beer’s criteria, was found to be associated with a functional decline at hospital discharge and at three months following discharge [13].

**Table 1:** Characteristics of the study population and according to number of potentially inappropriate prescription below or above median

Variable	All *n*= 461	PIP ≥4 *n* = 274	PIP < 4 *n* = 187
Age (years), m (SD)	77 (6.5)	78 (6.5)	76 (6.4)
Female, n (%)	254 (55.1)	153 (55.8)	101 (54.0)
Family situation, n (%)			
Married	219 (47.5)	118 (43.1)	101 (54.0)
Single	38 (8.2)	25 (9.1)	13 (7.0)
Divorced	61 (13.2)	35 (12.8)	26 (13.9)
Widowed	143 (31.0)	96 (35.0)	47 (25.1)
Living situation, n (%)			
Private household	419 (90.9)	247 (90.1)	172 (92.0)
Senior residency^a^	37 (8.0)	23 (8.4)	14 (7.5)
Retirement home or nursing home	5 (1.1)	4 (1.5)	1 (0.5)
Eucation, n (%)			
College qualification	130 (28.3)	74 (27.0)	56 (30.1)
Junior high school diploma	130 (28.3)	74 (27.0)	56 (30.1)
Secondary school diploma	187 (40.7)	120 (43.8)	67 (36.0)
No school degree	13 (2.8)	6 (2.2)	7 (3.8)
Statutory health insurance, n (%)	362 (78.9)	216 (79.1)	146 (78.5)
ISAR, m (SD)	2.8 (1.0)	3.0 (1.1)	2.6 (0.8)
Multimorbidity^b^, m (SD)	5.6 (3.6)	6.6 (3.8)	4.3 (2.8)
No. of hospital stays in last six months, m (SD)	0.8 (1.2)	0.8 (1.2)	0.7 (1.1)
Medications per participant, m (SD)	9.8 (4.5)	10.8 (4.5)	8.2 (4.1)
PIM per participant, m (SD)	1.7 (1.6)	2.4 (1.6)	0.6 (0.7)
PPO per participant, m (SD)	2.8 (1.5)	3.5 (1.4)	1.7 (0.9)
Quality of information: *good*, n (%)	406 (91.4)	243 (92.0)	163 (90.6)
Functional disability			
WHODAS cognition, m (SD)	21.3 (23.3)	25.1 (25.1)	15.8 (19.0)
WHODAS mobility, m (SD)	47.0 (32.2)	54.3 (31.5)	36.4 (30.3)
WHODAS self-care, m (SD)	25.6 (29.6)	32.3 (31.2)	15.8 (24.0)
WHODAS relationships, m (SD)	27.4 (26.8)	32.0 (28.7)	20.5 (22.2)
WHODAS life activities, m (SD)	51.7 (37.8)	59.3 (36.5)	40.7 (37.1)
WHODAS participation, m (SD)	40.0 (24.1)	44.8 (24.1)	33.4 (22.5)
WHODAS total score, m (SD)	34.9 (22.2)	40.5 (22.7)	26.9 (18.8)

*M* Mean; *SD* Standard deviation; *PIP* Potentially inappropriate prescription; *PIM* Potentially inappropriate medication; *PPO* potential prescribing omission; *ISAR* Identification of Seniors at Risk Screening Tool; *WHODAS* World Health Organization Disability Assessment Schedule 2.0 (0: no disability; 100: maximal disability); ^a^: senior residency: mainly independent living with minor nursing support; ^b^: Multimorbidity as measured by the outcome-orientated multimorbidity score “Activities of daily life” (ADL) of Tooth et al [34].

**Table 2:** Multiple linear regression analysis modelling PIM and PPO on functional disability measured using the WHODAS total score (*n*= 410, final model after variable selection)

	Reg. coeff. B	SE	95%-CI for B	Std. coeff. Beta	***p***-value
**No. of PIM**	2.65	0.57	1.53; 3.76	0.20	**< 0.001**
**No. of PPO**	1.49	0.64	0.24; 2.74	0.10	**0.020**
**ISAR**	5.27	1.01	3.28; 7.25	0.24	**< 0.001**
**Multimorbidity**^a^	0.84	0.29	0.26; 1.41	0.13	**0.004**
**No. of hospital stays in 6 m**	2.42	0.81	0.82; 4.01	0.13	**0.003**
**Gender, female**	6.55	1.82	2.96; 10.13	0.31	**< 0.001**
**Living independently**	-7.30	3.05	-13.30; -1.30	-0.35	**0.017**
**Quality of information: good**	-9.67	3.25	-16.06; -3.27	-0.46	**0.003**

*WHODAS* World Health Organization Disability Assessment Schedule 2 (with 0 points indicating no and 100 points indicating maximal functional disability); *Reg. coeff. B* Regression coefficient B; *SE* Standard error; *CI* Confidence interval; *Std. coeff. Beta* Standard coefficient Beta; *PIM* Potentially inappropriate medication; *PPO* Potential prescribing omission; *ISAR* Identification of Seniors at Risk Screening Tool. ^a^: Multimorbidity as measured by the outcome-orientated multimorbidity score “Activities of daily life” (ADL) of Tooth et al [32].

**Fig. 1:**
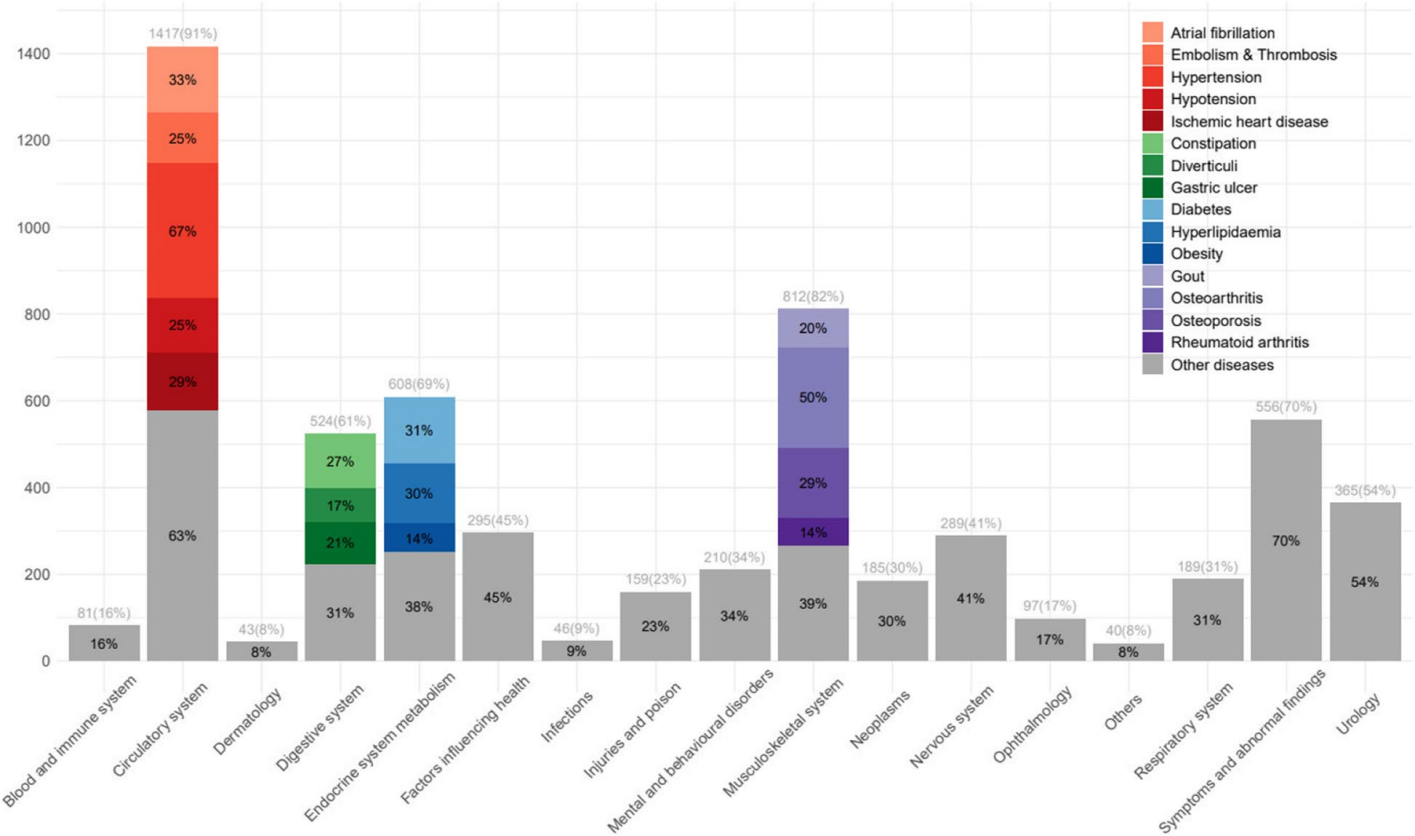
Total number of diagnoses according to ICD-10 classes. The numbers above each column indicate the total number of diagnoses according to ICD-10 class; the percentage indicates for the proportion of individuals with at least one diagnosis of the specific ICD-10-class. Note: “Symptoms and abnormal findings” correspond to ICD-10 class XVIII (R00-R99) and include symptoms not belonging to a specified disease [30]

**Fig. 2:**
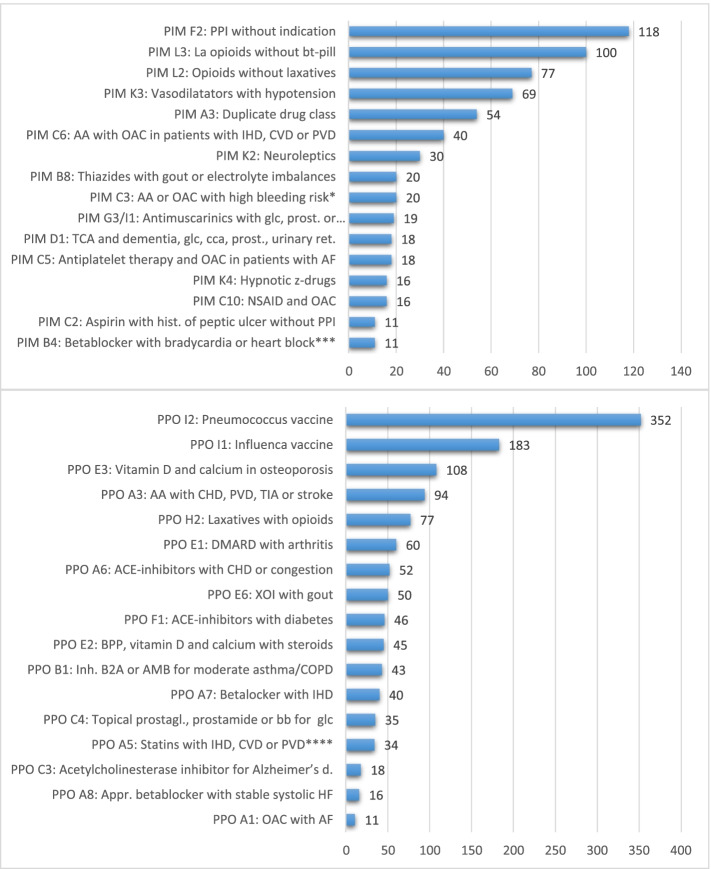
Frequencies of the potentially inappropriate medications (PIM) (above) and the potential prescribing omissions (PPO) (below) according to STOPP/START criteria version 2 [10] with a frequency above ten. PPI, proton pump inhibitor; la, long-acting; bt-pill, break-through-pill; AA, antiplatelet agents; OAC, oral anticoagulants; imb, imbalancies (here specifically: hypokalaemia, hyponatraemia, hypercalcaemia); IHD, ischaemic heart disease CVD, cerebrovascular disease; PVD, peripheral vascular disease; TCA, Ticyclic antidepressants; glc, glaucoma; CCA, cardiac conduction abnormalities; prost, prostatism; urinary retention; AF, atrial fibrilliation; TIA, transient ischemic attack; DMARD, disease modifying antirheumatic drugs; XOI, xanthine-oxidase-inhibitor, B2A, beta-2 agonist; AMC, antimuscarinic bronchodilatator; prostagl., prostaglandine; bb, betablocker; Alzheimer’s d., Alzheimer’s disease; appr, appropriate; HF, heart failure BPP, bisphosphonates. * High bleeding risk according to PIM C3 (STOPP/START criteria version 2 [10]): [documentation of] uncontrolled severe hypertension, bleeding diathesis, recent non-trivial spontaneous bleeding. ** PIM G3: Antimuscarinic bronchodilators with glaucoma or prostatism; PIM I1: Antimuscarinic drugs with dementia, glaucoma or prostatism; *** Type II or III heart block; **** unless the patient’s status is end-of-life or age is > 85 years

It must be acknowledged that polypharmacy, as well as PIMs, are only avoidable to a certain extent [14, 15], often leaving the prescriber with no alternative [16]. At the same time, polypharmacy can go hand in hand with under-prescribing or, more specifically, with ‘Potential Prescribing Omissions’ (PPOs) [17] that can be measured using the PPO-identifying items of the STOPP/START criteria [10, 18]. A longitudinal study in New Zealand, differentiating between Māori and non-Māori octogenarians, found an association between PPOs and mortality at 36-month-follow up in the Māori-group [19]. In a small cross-sectional analysis with 110 older participants PPOs were found to be associated with frailty [20]. Furthermore, a prospective examination of under-prescribing in outpatients with cardiovascular disease, also confirmed an association with under-prescribing and frailty [21]. However, overall, under-prescribing appears to be less well-documented in the recent literature.

Functional disability can lead to growing dependence, reduced quality of life, increased health care costs and augmented mortality in older patients [22–25]. Hence, functional disability has become recognised as an important patient-related outcome measure in clinical trials [26].

This study aims to investigate a potential association between PIMs as well as PPOs and functional disability in older patients at risk of further decline. Furthermore, the investigation seeks to characterise the population being studied and to assess the frequencies of PIMs and PPOs in this population.

## Methods

### Study design, setting and participants

This cross-sectional analysis was conducted as part of the LoChro trial, a randomised comparative effectiveness trial, to evaluate the effects of a local, collaborative, stepped and personalised care management approach for older people with chronic diseases and at risk of further functional decline in southern Germany [27]. People aged 65 years and older, at risk of further functional decline as indicated by a score of at least 2 of 6 points in the Identification of Older patients at Risk Screening Tool (ISAR) [28] were recruited from the inpatient and outpatient departments of the local university clinic between 2018 and 2020. The ISAR has demonstrated an association with unplanned readmissions, institutionalisation and the need for nursing care in older patients in Germany [29]. Exclusion criteria for the LoChro trial were: (1) terminal medical conditions, (2) lack of basic German-language skills or no German-speaking caregiver available and (3) not residing in the region [27].

### Variables and data sources

The following section describes the baseline-variables of the LoChro trial that were relevant to the present cross-sectional analysis. Age, sex, family situation, living situation, education, health insurance status, number of hospital stays in last six months, ISAR-score and the score of the World Health Organization Disability Assessment Schedule 2.0. (WHODAS) were assessed through interviews that were conducted with the older people and/or their family members or caregivers. Moreover, these interviews included a questionnaire asking for additional diagnoses relevant to the interpretation of the STOPP/START criteria Version 2.

Further diagnoses, medications, laboratory and other clinical parameters relevant to STOPP/START criteria Version 2 came from discharge letters, specialist reports in the case of outpatient visits, and other documents, such as laboratory results of the participating hospital. A team of two medical students, one junior and one senior practitioner, assessed diagnoses, medication, clinical data and the quality of available information. There was an expectation that the quality of data documented in the hospital system could vary. Thus, the quality of data available was allocated to two variables, with the expression “good” being used for current and complete diagnostic and medication data, and “sufficient” for data that included diagnostic and medication information documented in the six months prior to recruitment. Participants with no diagnostic or medication information in the last six months were excluded from the analysis. Diagnoses were defined according to the International Classification of diseases and related health problems, 10^th^ revision (ICD-10) [30], medication according to the Anatomical Therapeutic Chemical (ATC) classification system [31].

### Potentially Inappropriate Medication (PIMs), Potential Prescribing Omissions (PPOs), Multimorbidity and Functional Disability

PIMs and PPOs were defined by the STOPP/START criteria Version 2 [10]. Criteria that were not included were as follows: PIM A1, criteria that refer to the duration of treatment (PIM A2, H3,4,6) or treatment as first-line therapy (PIM B5,6, D2,12, F3, L1, PPO H1), and criteria that rely on special diagnostic issues due to a lack of differential information (PIM B7,9,12, D9,10, PPO A4, B3, C1,2,5, D2, E4,5, G1,2). PIM F2 was modified to “proton pump inhibitors without ongoing indication” whilst PIM D15, “neuroleptics without psychosis”, completed the catalogue following consensus within the medical working group.

The importance of confounding by multimorbidity in medication research has long been acknowledged [32]. However, counting the number of diagnoses within the hospital documentation system, and adding self-reported diagnoses, as measures of multimorbidity, are prone to bias due to overestimation. A recent systematic review of tools to measure multimorbidity for drug research recommended the use of indices validated for the outcome of interest [33]. Based on this proposal, Tooth et al.’s weighted multimorbidity index that is recommended for the outcome “activities of daily living” had been chosen to measure multimorbidity [34].

Functional disability was assessed by the World Health Organization Disability Assessment Schedule 2.0 (WHODAS) [35] combining the following criteria, validated in the German population: evaluation of cognition, mobility, self-care, relationship with others, life activities and participation in social life [36]. The WHODAS overlaps with common health-related quality of life measurements such as the Short Form Health Survey (SF-36) [37], and has been developed to provide a common measurement of disability, relying on the biopsychosocial model of the International Classification of Functioning, Disability and Health (ICF) [38, 39].

### Statistical methods

This examination included the characteristics of the study population presented through means and standard deviations, or absolute numbers and percentages, where appropriate. Characteristics of the groups below or above the median number of ‘Potentially Inappropriate Prescribing’ (PIP) (i.e. PIM plus PPO) were also presented. However, crude comparison tests were dispensed with, in order to minimise alpha error and false inferences. The frequency of diagnosis according to ICD-10 as well as the most frequently occurring PIMs and PPOs were illustrated using bar charts. Prior to regression analysis, crude correlations between explanatory variables were examined in order to compile the full regression model. The main explanatory variables (number of PIMs and number of PPOs) and the outcome factor (WHODAS-score) were considered as linear variables. Potential influences on the number of PIMs and PPOs on functional disability were then evaluated using multiple linear regression modelling. Backward variable selection, in which irrelevant variables are gradually eliminated from the full model, helped to obtain a stable final model in which the regression coefficients are unbiased and the associated p-values and confidence intervals are valid. Associations between the main explanatory and outcome variables were adjusted for age, sex, family status, living situation, education, health insurance (private vs. statutory), the ISAR score, weighted multimorbidity, number of regular medications, number of hospital attendances during the last six months, and the quality of information available.

The site of recruitment (inpatient vs. outpatient department) and the method of reporting (self-reporting vs. carer-reporting) were not included in the model, due to their *a priori* relationship with the number of hospital stays during the last six months, and multimorbidity. In addition, the requirements for linear analysis were examined. Analyses were performed using the open-source software *R*.

### Ethical considerations

All methods were performed in accordance with the relevant guidelines and regulations. Written informed consent was obtained from all participants or, if an individual had a legal representative, from the latter. The study was approved by the ethical committee of the University of Freiburg (Ethical vote 495/17).

## Results

At the time of analysis 484 participants were recruited for the LoChro trial and underwent baseline assessment. Data of 23 participants had to be excluded from the analysis due to insufficient information on diagnoses and medication. Characteristics of the remaining 461 participants and the subgroups of patients with the number of PIPs below or above median, are reported in Table 1.

The mean age was 77 (standard deviation [SD] 6.5) and 55% were female. Of the 461 participants only six (1.3 %) presented with no PIP. 122 (26.5%) had no PIM and 22 (4.8%) no PPO. On average participants received two PIMs (mean 1.7; SD 1.6; range 0-8) and would benefit from three more prescriptions (including vaccinations) (mean number of PPOs 2.8; SD 1.5; range 0-9). The mean WHODAS total score lay at 34.9 (SD 22.2) of 100 possible points, with 0 points indicating no disability and 100 points maximal disability. The mean number of medications taken was eleven (SD 4.7; range 0-23, median 10). Only 53 (11.5%) participants took less than five medications. The study population presented a high amount of multimorbidity as indicated by an average score of 5.6 (SD 3.6) in Tooth’s multimorbidity index and 2.8 (SD 1.0) in ISAR. The average number of diagnoses per participant was 13 (SD 5.0; range 2-25, median 12); ten (SD 4.3) of these were documented in the hospital documentation system.

A more in-depth picture of the study population’s morbidity is shown in the stacked bar chart of diagnoses in Fig. 1: Here, the number of diagnoses in the whole study population for each ICD-10-class is illustrated and the percentage of patients with at least one diagnosis of each specific ICD-10-class is given.

Frequent diseases within ICD-classes, such as ischaemic heart disease or hypertension, were marked separately. The top three ICD-10-classes were diseases of the circulatory system, musculoskeletal system and endocrine system. The most common diagnoses of the circulatory system werehypertension followed by ischaemic heart disease and atrial fibrillation. The most common diagnosis of the musculoskeletal system was osteoarthritis, whilst the most common endocrine disorder was Type II diabetes mellitus.

Figure 2 shows the frequencies of the PIMs and PPOs that occurred ten or more times in the study population. 17 PIMs were never observed in the population under study (PIM B2, B13, C1, C7, D6, D7, D11, E1, E2, E3, E5, F1, F4, G1, H8, J2, J6), 31 occurred one to ten times, only. PPO E7 was never observed and four PPOs (A2, B2, C6, D1) were observed between 1 and 10 times. The number one PIM was the prescribing of proton pump inhibitors without an ongoing indication. The most frequently reported prescribing omissions were pneumococcal and influenza vaccinations.

Participants with a number of inappropriate medications above median were often older, female, widowed, divorced or single compared to other participants. On average, patients with more than three PIPs scored higher in the multimorbidity score and took more regular medication. Furthermore, subjects in the group of inappropriate medication above median presented a significantly higher degree of functional impairment than others, as measured by an average WHODAS-score of 40.5 (SD 22.7) compared to 26.9 (SD 18.8) (difference in means 13. 6; 95%-confidence-interval [9.8; 17.4]; *p*-value < 0.0001).

Table 2 shows the results of the multiple linear regression analysis. After adjustment for all named possible confounders, the score of functional disability rose almost three points with one more PIM (regression coefficient B 2.7; 95%-confidence-interval 1.5 - 3.8), and 1.5 points with one more PPO (regression coefficient B 1.5; 95%-confidence-interval 0.2 – 2.7). Both associations were confirmed as statistically significant (*p* < 0.001 for PIM and *p* < 0.05 for PPO). The contribution of multimorbidity to the functional disability score was calculated as 0.8 (95%-confidence-interval 0.3-1.4; *p* < 0.005). Other significant contributors to functional disability were female sex, ISAR-scoring and number of hospital stays during the last six months. No significant contribution to the prediction of functional disability was observed for the number of medications. In total the model predicted 30% of the variance in functional disability (corrected R^2^ 0.28).

Regarding multicollinearity, all variance inflation factors were less than 1.5, and tolerance was greater than 0.5 for all variables. Residua were not correlated and their normal distribution, linearity and homoscedasticity were given.

## Discussion

In the present study of older patients at risk of functional decline, PIMs as well as PPOs demonstrated a significant association with functional disability. The modelled contribution of PIMs to the prediction of functional disability exceeded that of multimorbidity. Both, PIMs and PPOs contributed more to the prediction of the outcome than the number of medications.

Strikingly, PIMs and PPOs were common in the study population, with only six participants not experiencing any inappropriate prescribing according to STOPP/START criteria Version 2.

### Interpretation and Comparison to other studies

These results reflect the selection of older patients at risk: The LoChro-Study addresses older, chronically ill individuals; hence, our study population shows a high level of morbidity. On average, those being studied had an ISAR score of 2.9 (over two points indicating “seniors at risk of functional decline”) and took eleven regular medications. 408 (88.5%) of the participants took five or more medications, thus, fulfilling a common criterion of polypharmacy [1]. In the recently published analysis of the Survey of Health, Ageing and Retirement in Europe (SHARE), which included people aged 65 and above, 31% presented with five or more medications [40]. The morbidity of our study population also appears in the high average number of diagnoses. To analyse the appropriateness of medication in the present study, hospital data and self-reported diagnoses were combined, which led to more diagnoses overall. However, of the average number of diagnoses documented in the hospital documentation system, ten (SD 4.3), appeared comparable to the seven to ten diagnoses found in a sample of older patients with similar inclusion criteria in an Australian study [41]. In a German telephone health interview survey from 2009, 35% of people aged 75 and above suffered five or more conditions [42]. In 2012, the mean number of self-reported diagnoses in those aged 70 and above were three to four [43]. Our results are therefore not representative of all older patients in the country. However, they may be representative of older patients at risk of functional decline in Germany. Finally, the degree of functional disability is high in the sample studied. With a mean WHODAS-score of 35 points the average study participant lies on the 90^th^ percentile of the general population [35]. In conclusion, this survey deals with multimorbid, highly impaired, older patients.

The prevalence of PIMs and PPOs in this study are in accordance with other results: investigations into the prevalence of potentially inappropriate prescribing in acutely ill inpatients in six European hospitals found STOPP-listed PIMs in 35-77% participants and PPOs in 51-73% participants [44]. In a prospective study of under-prescribing in outpatients with cardiovascular disease in Germany, using STOPP/START V2 with a focus on cardiovascular medications alone, under-prescribing was common at 69% [21]. Furthermore, regarding the results of this study, the majority of participants were not vaccinated in accordance with national recommendations [45], osteoporosis prophylaxis was lacking, as well as medications for constipation and gout. Common PIMs referred to the safe use of opioids, orthostatic hypotension and neuroleptics without diagnosis. These observations were similar to a previous examination in the same region of Germany; only the use of Benzodiazepines, previously the most common PIM, appeared to have decreased, possibly indicating a positive effect of educational campaigns [46]. Pneumococcal vaccination rates amongst people aged between 60 and 72 in the German study region was assumed to be 13% [47], lower than 23% in this study of older patients. A recently published German study on influenza vaccination rates in older patients found a registered vaccination rate of 29-39% in the years 2011-2018 [48], considerably lower than our self-reported one-point survey (60%). Reporter and memory bias, especially in the case of pneumococcal vaccinations, and the selection of older patients at risk, might have supported these differences.

General Practitioners report feeling insufficiently trained in dealing with impairment and frailty amongst their patients, and see their role as dependent on patient motivation, and on the broader public health setting [49, 50]. Practitioners recognise that in daily consultations with older patients, the emphasis lies mainly on prescribing [49]. Whilst polypharmacy is consistently associated with physical decline [51], a recent study of over 2,500 individuals aged 65 and above found no association between PIMs and functional impairment, measured by a change in the Barthel-index at three-month follow up [8]. PIMs measured by the Beer’s criteria were found to be associated with a functional decline, in contrast to PIMs measured using the STOPP criteria in an Italian study [13]. A prospective cohort study confirmed a negative influence of PIMs on health-related quality of life [12]. However, several tools to identify PIMs have been published in order to meet the needs of a specific country, as well as the drugs marketed and the local prescribing culture. There may therefore be discrepancies in the outcomes assessed [52]. Furthermore, most of these studies were carried out in populations of older patients, where the variance of risk of loss of independence within the study group was disregarded [3, 8, 12, 13, 17]. Moreover, information on PIMs or PPOs was partly dispensed with by integrating them as binary variables [3, 13, 17, 21].

The results of our study indicate that a linear association between PIMs and PPOs, and functional disability, exists in highly impaired older patients.

Notably, the clinical relevance of a 1.5 or 2.7 increase, on average, in WHODAS score for PPOs and PIMs respectively, issupported by the minimal variation in WHODAS-scoring in the general population [35]. Interestingly, the prediction of WHODAS-score could be better explained by the number of PIMs than by a rise in multimorbidity score. In addition, the contribution of the number of medications to the variation in WHODAS score remained insignificant after inclusion of PIMs and PPOs. The latter strengthens the notion that it is not the quantity of medication that matters most, but the appropriateness of prescribing in the older patient.

The Cochrane Central Register of Controlled Trials lists a trial, that hypothesises an effect of optimised prescribing on the level of functional autonomy [53]. The findings of our cross-sectional study support the hypothesis that older patients who are at risk might benefit from interventions to reduce inappropriate prescribing.

### Strengths and limitations

A limitation of this study was that the cross-sectional design led to a more stable hypothesis, but it could not demonstrate a causal relationship. In addition, participants were recruited from different settings, so the quality of available data was diverse. However, this was controlled for in multiple linear regression analysis. Because not all STOPP/START criteria were used in the current study, the prevalence of PIP in the study population may have been underestimated. Furthermore, as medication lists originated partly from hospital discharge letters, the duration of medication effect on the patient needed to be considered. However, it was assumed that most of the over 4,500 prescribed medications in the study population had a prior influence on the participants.

The emphasis on older patients at risk of adverse outcomes acts as a strengthening factor between the chief explanatory and outcome variables. This study maintained a focus on older patients who had coped with polypharmacy, inappropriate medication and multimorbidity, Our results highlight how crucial appropriate prescribing is for such patients. In addition to information on diagnoses and relevant medication, laboratory results and further diagnostic parameters were also extracted, completing the picture of the morbidity of participants. To avoid a bias of over-diagnosis, a multimorbidity term was used to account for the possibility of confounding effects on the association of exposure and outcome variables. Finally, this study considers both PIMs and PPOs, thus presenting a holistic view of inappropriate prescribing and functional disability in the study population.

## Conclusion

The results indicate that interventions into inappropriate and under-prescribing might offer the opportunity to directly improve functional ability in older patients at risk of loss of independence. The frequency and clarity of the study findings show that these are actionable results with direct implications for daily practice.

## Data Availability

The datasets used and analysed during the current study are available from the corresponding author on reasonable request.

## Abbreviations

ATC: Anatomical Therapeutic Chemical
SF-36: Short Form Health Survey
ICF: International Classification of Functioning, Disability and Health
PIP: Potentially inappropriate Prescribing
SHARE: Survey of Health, Ageing and Retirement in Europe
bt-pill: Break-through-pill
AA: Antiplatelet agents
OAC: Oral anticoagulants
Abn: Abnormalities
CHD: Coronary heart disease
PVD: Peripher vascular disease
TIA: Transitory ischemic attack
DMARD: Disease modifying antirheumatic drugs
XOI: Xanthine-oxidase-inhibitor
BPP: Bisphosphonates
Reg. coeff. B: Regression coefficient B
SE: Standard error
CI: Confidence interval
Std. coeff. Beta: Standard coefficient Beta
